# Nanoscopic changes in the lattice structure of striated muscle sarcomeres involved in the mechanism of spontaneous oscillatory contraction (SPOC)

**DOI:** 10.1038/s41598-020-73247-1

**Published:** 2020-10-02

**Authors:** Fumiaki Kono, Seitaro Kawai, Yuta Shimamoto, Shin’ichi Ishiwata

**Affiliations:** 1 Department of Physics, Faculty of Science and Engineering, Waseda University, 3-4-1 Okubo, Shinjuku-ku, Tokyo, 169-8555 Japan; 2 Institute for Quantum Life Science, National Institutes for Quantum and Radiological Science and Technology, 2-4 Shirakata, Tokai-mura, Naka-gun, Ibaraki, 319-1106 Japan; 3 Laboratory of Physics and Cell Biology, Department of Chromosome Science, National Institute of Genetics, 1111 Yata, Mishima, Shizuoka 411-8540 Japan

**Keywords:** Muscle contraction, Skeletal muscle, Nanoscale biophysics, Structural properties

## Abstract

Muscles perform a wide range of motile functions in animals. Among various types are skeletal and cardiac muscles, which exhibit a steady auto-oscillation of force and length when they are activated at an intermediate level of contraction. This phenomenon, termed spontaneous oscillatory contraction or SPOC, occurs devoid of cell membranes and at fixed concentrations of chemical substances, and is thus the property of the contractile system per se. We have previously developed a theoretical model of SPOC and proposed that the oscillation emerges from a dynamic force balance along both the longitudinal and lateral axes of sarcomeres, the contractile units of the striated muscle. Here, we experimentally tested this hypothesis by developing an imaging-based analysis that facilitates detection of the structural changes of single sarcomeres at unprecedented spatial resolution. We found that the sarcomere width oscillates anti-phase with the sarcomere length in SPOC. We also found that the oscillatory dynamics can be altered by osmotic compression of the myofilament lattice structure of sarcomeres, but they are unchanged by a proteolytic digestion of titin/connectin—the spring-like protein that provides passive elasticity to sarcomeres. Our data thus reveal the three-dimensional mechanical dynamics of oscillating sarcomeres and suggest a structural requirement of steady auto-oscillation.

## Introduction

Muscle is a fibrous tissue that exerts contractile force for diverse motile functions, such as heartbeat and body movement, in animals. The force generation is associated with shortening of sarcomeres, which are the micron-sized cylindrical structures that are periodically arranged and connected in a series along the long axis of muscle fibers. Under physiological conditions, individual sarcomeres contract in response to the rise of free Ca^2+^ in the surrounding medium: they fully contract at a sub mM level of Ca^2+^ (i.e., on-state) and relax at sub µM level of Ca^2+^ (i.e., off-state). As a result, an iteration between the high and low Ca^2+^ levels can lead to, for example, a repeated contraction such as that required for cardiac pumping. On the other hand, sarcomeres can exhibit steady spontaneous oscillation of force and length at a fixed, intermediate level of Ca^2+^^1–5^. This phenomenon is called SPOC (Spontaneous Oscillatory Contraction)^6,7^. Although the molecular mechanism of SPOC has been extensively studied, it has not yet been completely clarified.

SPOC can be observed when sarcomeres are activated by Ca^2+^, or by a mixture of solution in which MgATP, MgADP, and inorganic phosphate (Pi) are present at certain concentration ratios in the absence of Ca^2+^^3,8,9^. The former was termed Ca-SPOC and the latter, ADP-SPOC^6,7,10^. In both cases, the activation is moderate and thus the force that develops in sarcomeres is below the maximal force level that can be achieved at a fully contractile condition. These two types of SPOC exhibit similar oscillation patterns of sarcomeres (as detailed below). Furthermore, (1) SPOC does not require cell membrane systems and thus it involves no local influx of Ca^2+^, and (2) the oscillation of a sarcomere propagates to adjacent sarcomeres at a speed which can be faster than the diffusion of chemical substances. Taken together, SPOC is the property inherent in the contractile system but not attributable to the oscillation of chemicals. The detailed characteristics of SPOC have been summarized in several review articles including ours^6,7,10–12^.

In the myocardium, the period of the sarcomere oscillation is strongly correlated with the resting beating rate of the heart in diverse animal species, including rats, rabbits, dogs, pigs, and cattle. The correlation can be found in both ADP-SPOC^13^ and Ca-SPOC^4^. Our recent studies further showed that myofibrils prepared from human myocardium can exhibit Ca-SPOC, with an oscillation period that matches the resting heart rate of humans^12,14^. Despite the yet-to-be-determined causality, these correlations infer a physiological role of SPOC in the heartbeat mechanism. Moreover, cardiac myofibrils are not fully activated even at systole of the heart; the concentration of free Ca^2+^ is maintained relatively low (~ 10^−6^ M) (cf. Refs.^15,16^), to a level similar to the Ca-SPOC condition.

Apart from several theoretical models, a series of models that we recently developed can recapitulate essentially all the characteristics of SPOC (i.e., the “unit model” for single sarcomeres^17^; the “series-connected model” for single myofibrils^18^; the “bundled model” for small bundles of myofibrils^19^). In particular, our models can reproduce the following four oscillatory patterns that are the primary characteristics of SPOC: (1) in-phase synchronization (all sarcomeres oscillate synchronously across a myofibril so that the amplitude of tension oscillation of a myofibril is largest among several SPOC patterns), (2) metachronal traveling waves (the lengthening phase of a sarcomere propagates to the adjacent sarcomeres one by one along a myofibril), (3) disrupted traveling waves (traveling waves appear at multiple locations in a myofibril and disappear when the wave-fronts collide), and (4) out-of-phase synchronization (individual sarcomeres in a myofibril oscillate randomly with no synchronous movement, consequently the tension oscillation of the myofibril is relatively minor).

A key assumption implemented in our models is that the probability of cross-bridge formation depends on the nanoscale spacing between the thin (actin) and the thick (myosin) filaments in sarcomeres, which changes as a function of sarcomere length (SL) that oscillates in SPOC. Specifically, the longer the SL, the narrower the myofilament lattice spacing and thus the greater the probability of cross-bridge formation. This assumption is supported by several experimental findings demonstrating that the filament lattice spacing decreases with increasing SL, especially when the muscle is in a relaxed state^20–24^. It has been proposed that this SL-dependent change in the filament lattice spacing is directly involved in the control of the magnitude of contractile force in muscle (cf. Ref.^25^). At the maximum level of activation condition, the filament lattice spacing is nearly independent of the SL, and therefore, the amount of developed force becomes proportional to the extent of overlap between the thick and thin filaments, which increases with decreasing SL. This provides the basis of the sliding filament theory for muscle contraction^26^. On the other hand, at intermediate levels of activation condition, the relationship between the force and the filament overlap length becomes non-linear^5,27–30^, with which sarcomeres generate less force and become mechanically unstable as the SL shortens. Despite several indirect evidence, whether the myofilament lattice dynamically oscillates during SPOC remains unclear.

Titin/connectin^31–33^ is a long, thread-like protein running along each sarcomere (for a review, see Ref.^34^). This protein connects sarcomere’s Z-disk and M-band and can generate an elastic restoring force, which can contribute to the longitudinal force balance in sarcomeres (e.g., Ref.^35^). Also, titin/connectin mediates a lateral interaction between thick and thin filaments^36,37^ and can narrow the myofilament lattice spacing when sarcomeres are extensively stretched^38–41^. Although our SPOC models^17–19^ do not require the passive elastic properties of titin/connectin, some other models suggest their predominant contribution (e.g., Ref.^42^). The role of titin/connectin in SPOC is thus controversial and needs to be experimentally confirmed.

In this paper, we test the above-mentioned predictions by analyzing the nanoscopic change of sarcomeres in SPOC. Specifically, we measure the change in sarcomere width by using tracer microbeads attached to the lateral sides of a sarcomere’s A-band. We demonstrate that the width of the sarcomere’s A-band (Aw) oscillates at an anti-phase with the oscillation of SL. These findings, which are based on our previous works^43,44^, agree with a recent report analyzing the phase-contrast image of a sarcomere^45^. We also show, using a proteolytic digestion of titin/connectin, that this elastic protein plays a role in modulating the oscillatory pattern of SPOC, but it is not a pre-requisite for the generation and maintenance of auto-oscillation. Together, our data underpin the significance of the longitudinal and lateral force balance in sarcomeres for SPOC and propose the possible molecular mechanisms.

## Materials and methods

### Preparation of myofibrils

All procedures conformed to the Guidelines for Proper Conduct of Animal Experiments approved by the Science Council of Japan, and were performed according to the Regulations for Animal Experimentation at Waseda University with the approval of the Committee for Animal Experiments at Waseda University. Glycerinated muscle fiber was prepared from psoas of white male rabbits (2.5–3.0 kg, conventional). Rabbits were anesthetized by intravenous injection of sodium pentobarbital (25 mg/kg) to the ear. After decapitation, the body was dissected and placed on ice for 30 min. Then, the psoas muscle was cut into strips (5–7 cm long and 3–5 mm thick), and fixed at both ends onto a glass rod of 3 mm in diameter using a thin cotton thread. The muscle fibers mounted on glass rods were soaked in glycerol solution [51% (v/v) glycerol, 0.5 mM NaHCO_3_, and 5 mM EGTA], stored at 2 °C for 24 h, and then stored at − 20 °C until required after replacing the solution with a fresh glycerol solution.

Single myofibrils and small bundles of myofibrils were prepared by homogenizing the glycerinated psoas fibers as described previously^46^, except that leupeptin was added to the glycerol solution to suppress the proteolysis of samples^36^. Glycerinated muscles with a storage period of 7–80 days were used in this study. Seven days were required for the glycerol solution to penetrate the muscle fibers.

### Solutions

The following solutions were used unless otherwise stated: relaxing solution, 0.7 mM free Mg^2+^, 3.3 mM MgATP, 20 mM 3-(N-morpholino)propanesulfonic acid (MOPS), 4 mM EGTA, Ionic Strength (I.S.) 160 mM (KCl) and pH 7.0 (KOH); Rigor solution, 2 mM free Mg^2+^, 20 mM MOPS, 4 mM EGTA, I.S. 150 mM (KCl) and pH 7.0 (KOH); SPOC solution, 1.4 mM free Mg^2+^, 0.2 mM MgATP, 2 mM MgADP, 4–8 mM inorganic phosphate (Pi), 20 mM MOPS, 4 mM EGTA, I.S. 160 mM (KCl) and pH 7.0 (KOH). In experiments examining the effect of osmotic pressure, dextran T-500 (average mol wt 400,000–500,000, D1037, Sigma-Aldrich, St. Louis, MO, USA) was used at varying concentrations. In experiments for proteolysis of titin/connectin, trypsin was diluted in a relaxing solution and used at a concentration of 0.10 µg/ml. Leupeptin was used at a concentration of 100 µg/ml. Polystyrene microbeads were used after diluting 700–1500 times in the rigor or relaxing solutions.

ATP and ADP were purchased from Roche Applied Science (Indianapolis, IN, USA); trypsin was from Sigma-Aldrich (from bovine pancreas, T8003). Other chemicals were of reagent grade. Polystyrene microbeads were purchased from Polysciences (diameter: 0.535 ± 0.01 µm, 07307, Warrington, PA, USA).

### Experimental setup

When the tension generated by myofibrils was measured, a single myofibril or a thin bundle of myofibrils was held with a pair of glass microneedles in the rigor solution according to the previously described method^47^. The microneedles were made of glass rods (1 mm in diameter, G-1000, Narishige, Tokyo, Japan) using a pipette puller (PB-7, Narishige). The elastic constant of the flexible microneedle was determined by the cross-calibration method^47^. The motion of microneedles was controlled by using hydraulic three-axis micromanipulators (MHW-3, Narishige) mounted on an inverted phase-contrast microscope (TE2000, Nikon, Tokyo, Japan). One of the microneedles had a 50-fold stiffer tip than the other (the elastic constant of the flexible microneedle’s tip was 3.9–14.6 nN/µm). Myofibrils containing 15–20 sarcomeres were used in the experiments. A myofibril was suspended in a chamber and the solutions were exchanged by a peristaltic pump (SJ-1211, ATTO, Tokyo, Japan). A YAG laser (iPG Photonics Japan, Yokohama, Japan) was used as the optical tweezers for trapping microbeads. All experiments were carried out at 25 ± 2 °C.

Phase-contrast images of myofibrils were acquired using 40 (or 100) × objective lenses (PlanFluor ELWD 40×, PlanFluor 100×, Nikon) and a high-speed camera (FASTCAM-1024PCl, Photron, Tokyo, Japan). The images were recorded on a computer using the Photron FASTCAM Viewer (Photron) at the sampling rate of 30 or 60 fps. In the experiments with dextran, phase-contrast images were recorded on a digital video (DV) through a CCD camera (CCD-300, Dage-MTI, Michigan City, IN, USA) at the sampling rate of 30 fps. For the recording, the orientation of the CCD camera was adjusted so that the myofibril long axis could be aligned parallel to the horizontal axis of the camera.

In some experiments, myofibrils that were adhered to the glass surface, and not those suspended between a pair of microneedles, were analyzed. The myofibrils of relatively long lengths with ends firmly attached to the glass surface, but no significant friction in the central part, were chosen for analyses. In these experiments without microneedles, another phase-contrast microscope (TMD300, Nikon) system was also used, as described previously^48^.

### Effects of dextran

It is known that when the polymer dextran is added, water is excluded from the muscle by osmotic pressure and the lattice spacing is reduced (cf. Refs.^49–51^). To examine the effects of dextran on SPOC, the rigor solution was replaced with a SPOC solution containing varying dextran T-500 concentrations (0–1%). The measurement was repeated by exchanging solutions several times (e.g., 0% → 0.5% → 1% → 0.5% → 0%) and completed before the SPOC pattern became disorganized.

### Tryptic treatment of myofibrils

The effects of tryptic treatment on the resting tension of myofibrils and on the oscillatory pattern of SPOC were examined as follows: first, both ends of myofibrils were fixed to glass microneedles in the rigor solution^47^. Then, the solution was replaced with the rigor solution containing plastic beads, which were attached to lateral sides of the A-band (near the M-band) of a sarcomere by using optical tweezers. The buffer solution was then replaced with the relaxing solution (for resting tension measurements) or the SPOC solution (for oscillation measurements). In the former case, the myofibril at some SL was treated with 0.10 µg/ml trypsin for 2 min, and then trypsin activity was inhibited with 100 µg/ml leupeptin. The resting tension was measured during this process. And then, the myofibril was stretched to extend the sarcomeres by moving the stiff microneedle, and the resting tension, the average SL, and the A-band width (Aw) were measured. This procedure was repeated so that the relationship among the SL, Aw, and the resting tension, and the effects of tryptic treatment on these parameters could be examined. In the case of measuring the effects of tryptic treatment on the SPOC properties, the same procedure as described above was applied. SL, Aw, and tension oscillation patterns were recorded throughout the tryptic treatment.

Fluorescence imaging of titin/connectin in myofibrils was performed by using a monochronal anti-titin antibody (ab7034, Abcam, Cambridge, UK; clone T11; recognizing 0.05 µm from the end of the A-band^52^) labeled with fluorescent dye (Z25260, Invitrogen, Waltham, MA, USA) according to the manufacturer’s protocol. After the tryptic treatment, the myofibrils were thoroughly washed with relaxing solution containing 100 µg/ml leupeptin, and then incubated with the dye-labeled antibody for 20 min. Imaging was performed using a Hg lamp with an excitation filter (550 nm) and a long-pass emission filter (590 nm cut-off) and a high-sensitivity ICCD camera (ICCD-350F, Video Scope International, Dulles, VA, USA). The phase-contrast image of the corresponding myofibrils was acquired using a different light path equipped in the microscope, via a CCD camera (CCD-300, Dage-MTI). Non-reactive antibodies were washed out before imaging. The entire procedure was carried out at room temperature (25 ± 2 °C).

### Data analysis

**Figure 1:**
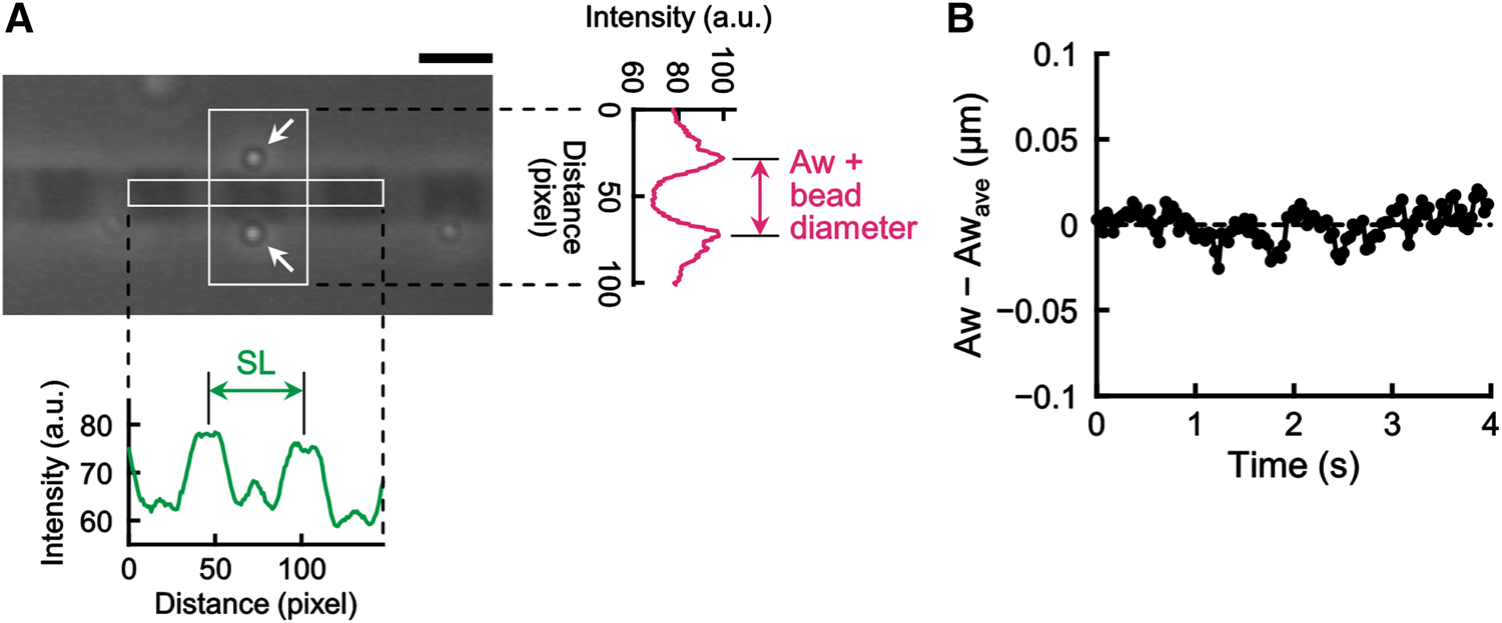
Method and validation for SL and Aw measurements. (**A**) Phase-contrast image showing the measurements of SL and Aw in a single skeletal myofibril. A pair of tracer microbeads are attached to the lateral of the A-band center, i.e., M-band. Line scans outside the micrograph show intensity profiles generated along the long (green) and short (pink) myofibril axes. Scale bar 2 µm. (**B**) Accuracy of microbead position measurement. Fluctuation of microbead positions at the A-band in the relaxing condition.

Phase-contrast images of myofibrils were saved on a computer in TIFF format. Scion Image (Scion Corporation, Frederick, MD, USA) was used to obtain the intensity profiles of myofibrils along the long fibril axis at each time point, which were then analyzed using a self-written macro in Microsoft Excel (Microsoft, Redmond, WA, USA) to obtain the intensity peak positions. The distance between adjacent I-bands, which appeared as bright, narrow intensity peaks in the profile, was defined as SL (Fig. 1A). The frequency analysis of SL oscillation was performed using PowerLab Chart 5 (AD Instruments Japan, Nagoya, Japan).

To determine the A-band width (Aw) of a sarcomere, the distances between the brightest peaks of the two tracer microbeads attached to each A-band were measured by analyzing the centroid position of each microbead, and subtracted by 0.535 µm, which corresponds to the diameter of each microbead (Fig. 1A).

Tension of myofibrils was estimated by multiplying the displacement of flexible glass microneedle by its elastic constant. Because of the small elastic constant of the flexible microneedle, the total length of myofibril oscillated by about 20% during SPOC, which means that the force measurement was performed in an auxotonic condition, i.e., the condition at which both the generated force and the total length of the myofibril can change simultaneously.

## Results

### Establishing a method to measure changes in the sarcomere’s three-dimensional lattice structure

**Figure 2:**
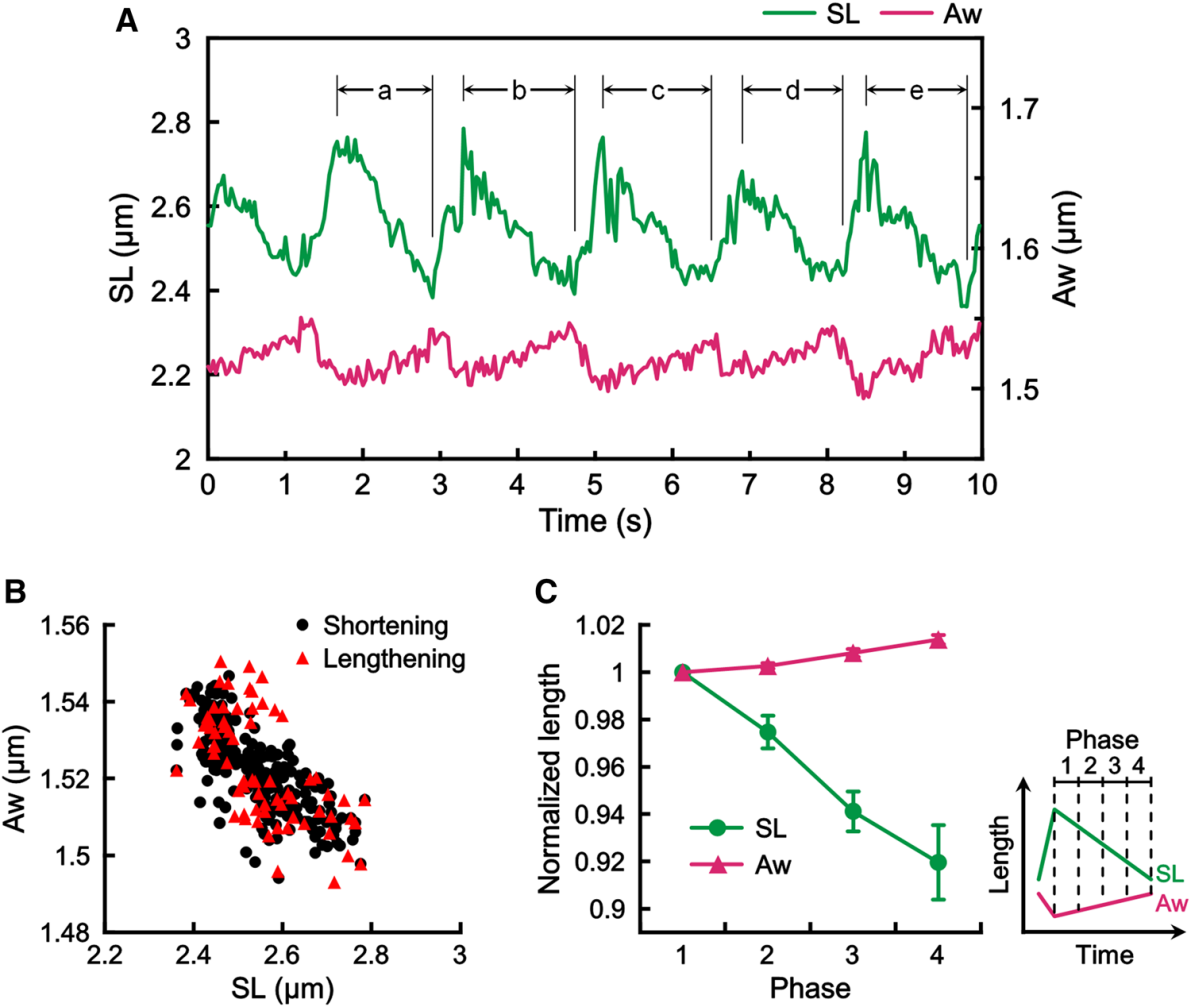
The Aw value of sarcomeres oscillates anti-phase with SL in SPOC. (**A**) An example showing the time course of the changes in SL (green) and Aw (pink) of a single sarcomere during SPOC. (**B**) Correlation between the changes in the SL and the Aw within the sawtooth wave form in SPOC. The data in the shortening phase [corresponding to the regions a–e in (**A**), black circles] and the lengthening phase [the regions between the shortening phases in (**A**), red triangles] are separated. (**C**) Changes in SL and Aw of a sarcomere during the shortening phase of SPOC. The shortening phase was further divided into 4 phases equally as depicted in the schematic at the bottom right. The SL (green) and the Aw (pink) were averaged in each phase and normalized as 1 for phase 1 [n = 5 shortening phases in (**A**), error bars, SD].

**Figure 3:**
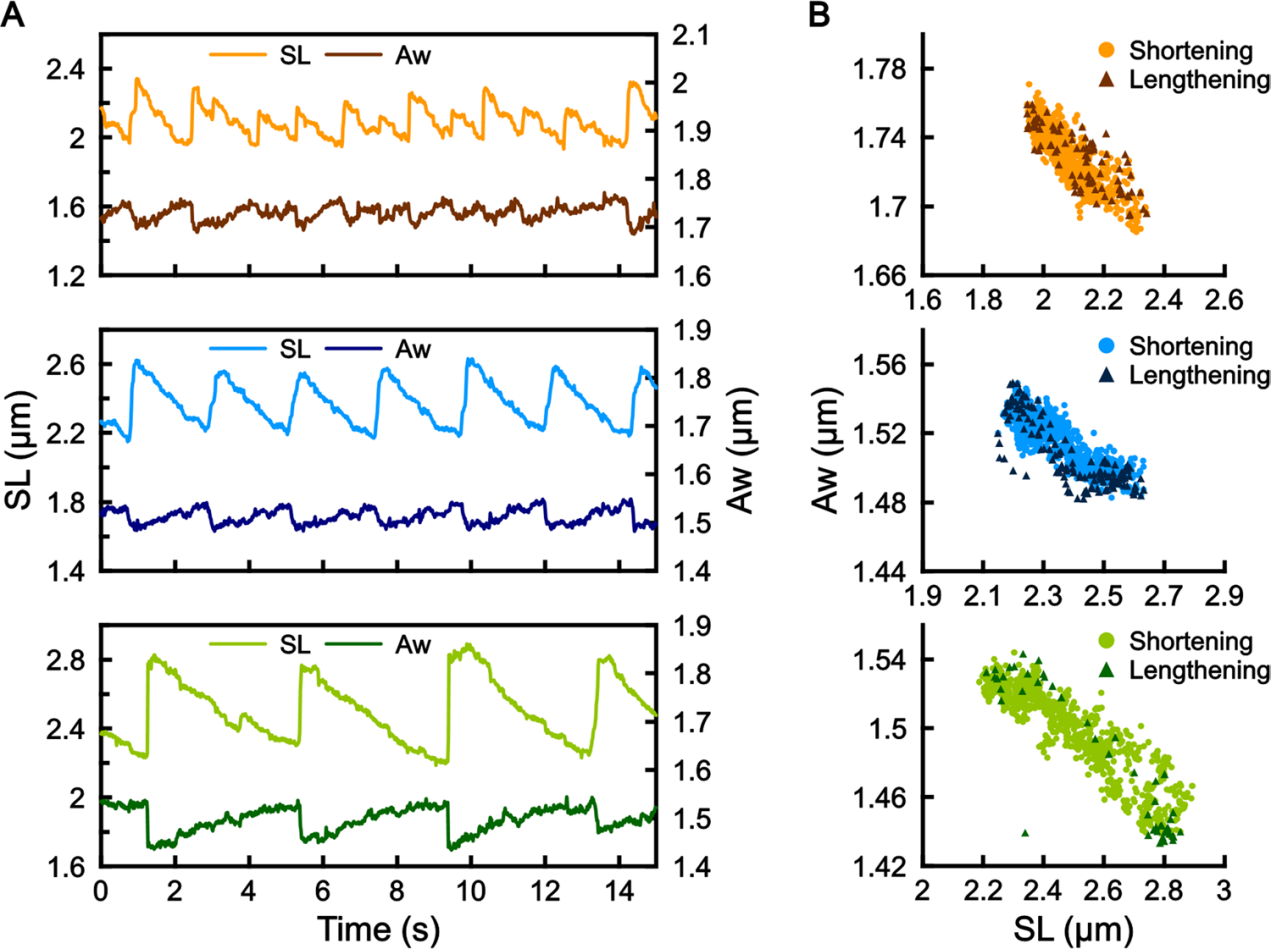
The Aw value of sarcomeres oscillates over a range of average SLs in SPOC. Both ends of myofibrils were held with glass microneedles to examine SPOC. All three graphs were obtained from the same sarcomere in a myofibril. The average SLs are 2.10 µm (upper), 2.36 µm (middle) and 2.51 µm (bottom). The myofibril was stretched stepwise from the top to the bottom. (**A**) Time course of the changes in SL and Aw obtained at the video rate of 60 fps. (**B**) Correlation between the changes in the SL and the Aw within the sawtooth wave form of sarcomeres in SPOC. The data were obtained from the corresponding figures in (**A**).

We sought to establish a method that allows us to quantitatively analyze the changes in the three-dimensional structure of sarcomeres at a high spatiotemporal resolution. Phase-contrast imaging revealed a clear striation pattern of sarcomeres along the long axis of myofibrils; SL could be determined by measuring the distance between the adjacent intensity peaks that appeared in a line-scan profile (green trace and double-arrow, Fig. 1A). The A-band width (Aw) is known to correlate with the lattice spacing of myofilaments^50,53^. However, analysis of the Aw using the phase-contrast image is not straightforward because no optically distinct structure exists along the short axis of myofibrils. To this end, we took an alternative approach, by attaching a pair of tracer microbeads to the sides of single sarcomeres by using facile nanomanipulation of optical tweezers; the distance between the pair of the microbeads was measured (white arrows, Fig. 1A) [Aw was defined as the value after subtraction of the bead diameter]^29^. The magnitude of the Aw fluctuation, which was measured in a relaxing condition and predominated by the Brownian movement of individual microbeads, was 8.0 ± 1.4 nm (mean ± SD, n = 3) (Fig. 1B). The magnitude of this Aw’s basal “noise” was significantly smaller than the magnitude of Aw oscillation experimentally observed during SPOC (cf. Figs. 2, 3) and the one predicted by our model.

### The A-band width (Aw) oscillates in an anti-phase with sarcomere length (SL) in SPOC

In establishing the measurement technique described above, we analyzed the dynamic changes of SL and Aw in SPOC (Fig. 2; Supplementary Movie S1). To do so, a single myofibril that exhibited a stable SPOC pattern was first selected and then microbeads were attached (as described above) to a sarcomere after pausing the oscillation by flushing a relaxing solution. Then, the myofibril was exposed to the SPOC solution again, so that the oscillation was resumed. As shown in Fig. 2A (red trace), we found that the Aw of the sarcomere in SPOC gradually increased as SL shortened (green trace, Fig. 2A); the Aw narrowed as the SL increased. This anti-phase relationship between SL and Aw was consistently observed over the repeated cycles of oscillation in single sarcomeres and across several myofibril samples (n = 8). Plotting the Aw as a function of SL revealed a strong negative correlation for both shortening and lengthening phases of the sarcomere oscillation (Fig. 2B). Averaging the SL and Aw data at four equally-divided phases of the oscillation revealed that the amplitude of the Aw oscillation was ~ 2% while SL changed by ~ 8% (Fig. 2C). This corresponds to the Aw oscillation size to be ~ 40 nm, which was well above the limit of our spatial resolution (~ 8 nm; Fig. 1B) and comparable to the value predicted from our theory^17^. Moreover, the properties of SL oscillation, such as the period, amplitude, and the travelling pattern, observed in myofibrils with microbeads attached were comparable to the typical properties of SPOC observed without microbeads. Together, these results indicate that the Aw periodically changes coupled with SL oscillation in SPOC.

### The A-band width (Aw) oscillation occurs over a range of average sarcomere length (SL) in SPOC

SPOC occurs at a range of average SL from ~ 2.2 to 3.0 µm, which changes depending on the amount of load applied to the myofibril. To examine whether the Aw oscillation can be observed at varying average SLs in SPOC, we held the ends of a single myofibril by a pair of microneedles using a previously established method^5,14,29,47,54^ and stretched the myofibril by altering the distance between the two microneedles. A pair of microbeads was attached to the lateral sides of a sarcomere’s A-band prior to the measurement. As shown in Fig. 3, we observed a clear oscillation pattern of Aw over a range of average SLs. At a short average SL (~ 2.1 µm), the amplitude of SL oscillation was relatively small and the period was not perfectly ordered, as reported previously (e.g., Fig. 1f in Ref.^18^); the Aw oscillation follows the pattern of the relatively disorganized SL oscillation (Fig. 3A, top panel). At a middle range of average SL (~ 2.4 µm), we observed a more periodic, sawtooth pattern of SL that was tightly coupled in anti-phase with the Aw oscillation (Fig. 3A, middle panel). At a long average SL (~ 2.6 µm), the amplitude and the period of SL oscillation became larger compared to those at shorter average SLs, and likewise the oscillation amplitude of Aw increased (Fig. 3A, bottom panel). In either case, the change in the SL was tightly coupled with the change in the Aw, and accordingly, they exhibited a strong negative correlation (Fig. 3B). Therefore, we conclude that the anti-phase oscillation of SLs and Aws are the well-conserved property of SPOC.

### Effect of dextran on the oscillatory patterns of SPOC

Given the prominent change of Aw in SPOC, we next investigated whether and how the myofilament lattice spacing affects SL oscillation. In particular, we focused on the fact that the average Aw during oscillation decreases with increasing SL. We hypothesized that a reduction in the average myofilament lattice spacing results in a change in SL oscillation. To test this, we osmotically compressed the myofilament lattice of sarcomeres by supplementing the macromolecule dextran.

**Figure 4:**
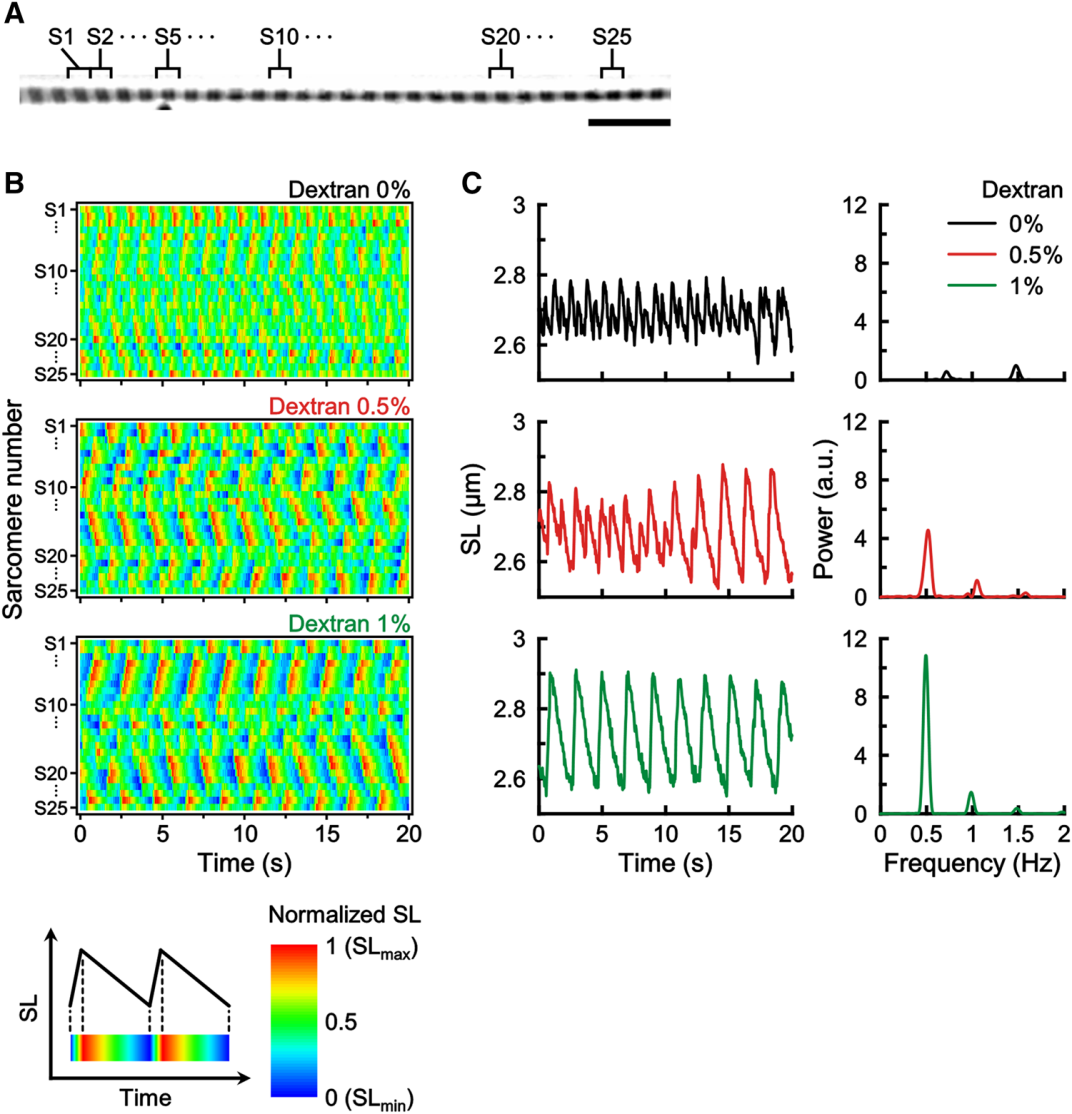
Effect of dextran concentrations on the SPOC pattern. The dextran concentration was stepwise increased from 0 → 0.5 → 1.0% for the same myofibril. (**A**) A phase-contrast image of a myofibril in which the changes of SLs were analyzed. The sarcomeres analyzed are numbered S1–S25. [The black shadow under S5 is a piece of debris attached to the myofibril.] Scale bar 10 µm. (**B**) Two-dimensional representation of the time series of sarcomere oscillations in a single myofibril. The amplitude of SL oscillation is shown in color, which was normalized in each oscillation as shown below. (**C**) The time course of SL oscillations for the sarcomere number S4 (left) and their respective power spectrum (right).

We found that the SL oscillation, which was relatively unstable in the absence of dextran (Fig. 4B,C, top panels), became more stable as the dextran concentration increased (correspondingly, the average lattice spacing presumably decreased) (Fig. 4B,C, middle and bottom panels). The effect of dextran was reversible. That is, the stable SL oscillation appeared every time the solution was exchanged to 1% dextran, as 1% → 0% → 1%. Similarly, the unstable SL oscillation appeared every time the solution was exchanged back to 0% dextran.

### Changes of SPOC patterns associated with the change in myofilament lattice spacing

**Figure 5:**
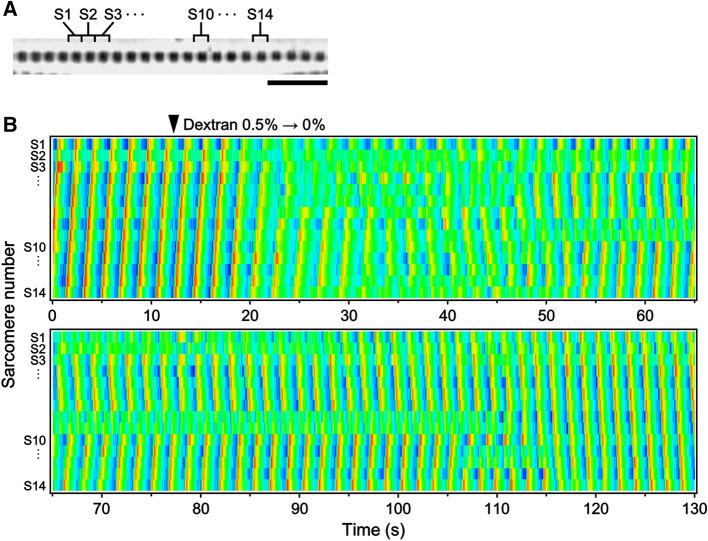
An example showing a SPOC pattern temporarily changed by a change in dextran concentration. (**A**) A phase-contrast image of a myofibril in which the changes of SLs were analyzed. The sarcomeres analyzed are numbered S1–S14. Scale bar 10 µm. (**B**) The dextran concentration was changed from 0.5 to 0% at the time shown by an arrowhead. The amplitude of SL oscillation is shown in color, as shown in Fig. 4B bottom.

We also analyzed whether the travelling wave of SPOC can be modulated by changing the lattice spacing of sarcomeres. As shown in Fig. 5, immediately after increasing the lattice spacing with the removal of dextran (0.5–0%), the pre-organized sarcomere oscillation (metachronal SPOC wave) was transiently disrupted and then a stable travelling wave gradually re-appeared. In the transient state, the sarcomere oscillation was disordered locally, and as time progressed, the oscillatory phases of adjacent sarcomeres started to synchronize and finally the metachronal SPOC wave was re-established. These results indicate that the myofilament lattice spacing can influence the pattern of sarcomere oscillation but the travelling wave of SPOC is robust against its change.

### Examining the effect of tryptic treatment on titin/connectin in sarcomeres

**Figure 6:**
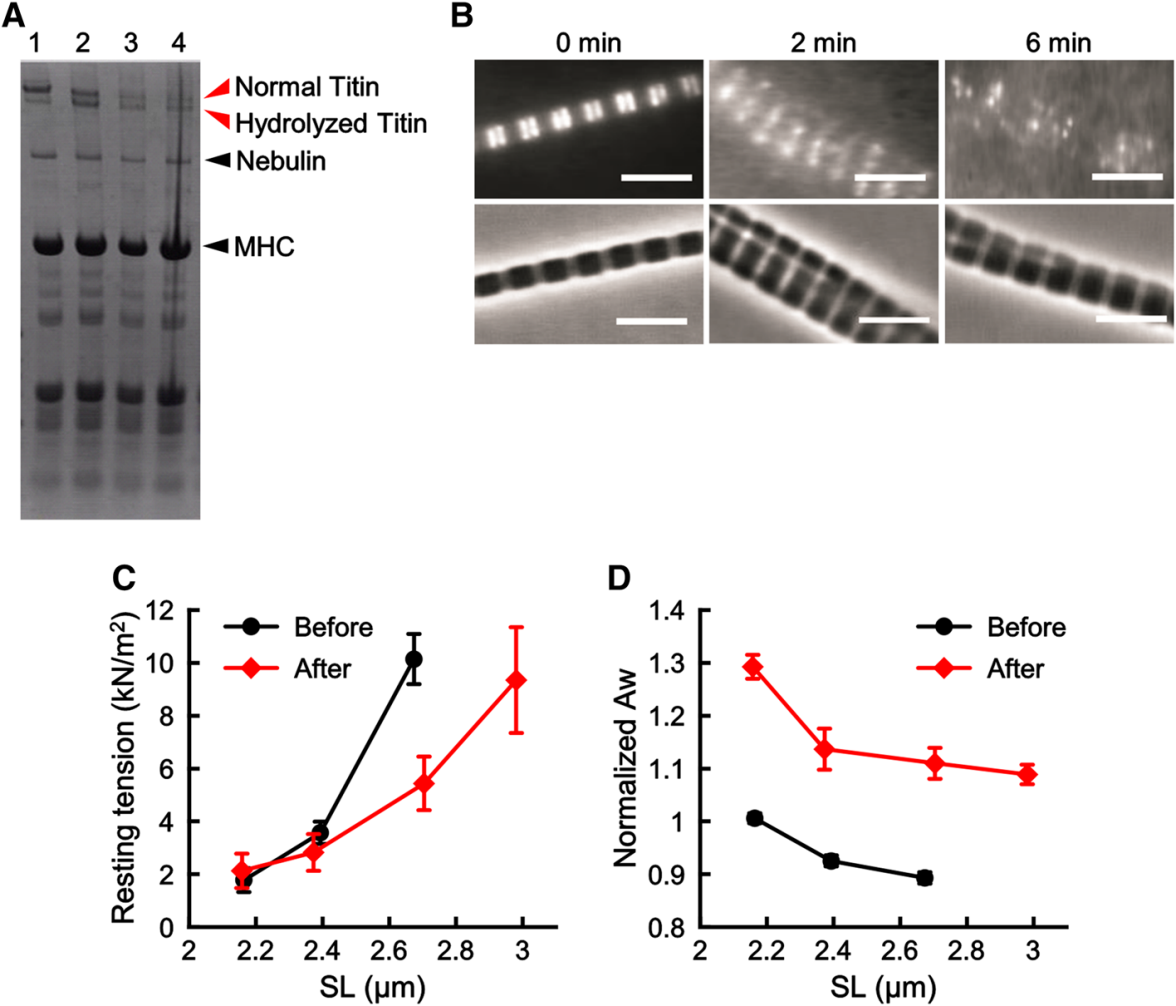
Examining the effect of tryptic treatment on the structure and the contractile properties of myofibrils. (**A**) SDS-PAGE of trypsinized muscle fibers. Lane 1: untreated, lane 2: treated for 2 min, lane 3: treated for 4 min, lane 4: treated for 8 min. The gel image was cropped and flipped horizontally. For the original full-sized image, see Supplementary Fig. S1. (**B**) Fluorescence images (upper) and phase-contrast images (lower) of myofibrils before and after the trypsinization. Trypsinization times were 0 min, 2 min and 6 min (from the left to the center and the right, respectively). After tryptic treatment for each period indicated, the protease reaction was halted by washing myofibrils with leupeptin and then fluorescence staining was employed using a dye-labeled antibody to titin/connectin (excitation at 555 nm and emission at 565 nm). The micrographs are an accumulation of 10 video still images to reveal the relatively weak signal at 6 min. For each myofibril presented, fluorescence and phase-contrast images were taken using different cameras (see “Materials and methods”) and their x–y positions were approximately aligned. Scale bars 5 µm. (**C**) Effect of tryptic treatment on the resting tension at several SLs (n = 5, error bars, SEM). The ends of myofibrils were held with glass microneedles to examine the resting tension at different SLs. The data on the SL dependence were obtained from the same myofibril. The value at each SL is the average of 5 different myofibrils. Black and red symbols, before and after 2 min of tryptic treatment, respectively. (**D**) Effect of tryptic treatment on the relative value of Aw at several SLs (normalized at SL = 2.15 µm before the tryptic treatment) (n = 5, error bars, SEM). The data were obtained from the same experiments as shown in (**C**). Black and red symbols, before and after 2 min of tryptic treatment, respectively.

Titin/connectin is known to play a role in (1) generating an elastic restoring force along the long axis of myofibrils, and (2) altering the myofilament lattice spacing in a SL-dependent manner^34^. To examine the possible roles of titin/connectin in SPOC, we treated myofibrils with trypsin, a serine protease that can preferentially digest titin/connectin^55–57^. An SDS-PAGE analysis of muscle fibers treated with 0.10 µg/ml of trypsin demonstrated that titin/connectin almost disappeared after 4 min of treatment (red arrowheads, Fig. 6A). In contrast, the integrity of other proteins was not significantly altered by this treatment.

To further examine the impact of tryptic treatment on the titin/connectin at the sarcomere level, we stained myofibrils with a fluorescent-labeled monoclonal antibody to titin/connectin and analyzed the change in the signal intensity upon tryptic treatment (Fig. 6B) (see “Materials and methods”). To maintain the integrity of the antibody, myofibrils were pre-washed with a solution containing leupeptin (100 µg/ml), a serine protease inhibitor, before staining. We found that, with the same trypsin dosage and time period used for muscle fibers (Fig. 6A), the fluorescence signal was significantly weaker in the treatment time of about 2 min compared to 0 min control (Fig. 6B, middle versus left panel). On the other hand, the overall myofibril structure, as observed in phase-contrast images, did not show noticeable changes until the tryptic treatment exceeded 6 min (Fig. 6B, right panel). The resting tension of myofibrils, which was measured in a separate assay by stretching the two ends of myofibrils using a pair of force-calibrated microneedles, gradually decreased and reached ~ 1/2 of the initial value at 2 min from the start of the tryptic treatment (Fig. 6C) before it reached a nearly zero value at 4 min (dosage: 0.10 µg/ml). The addition of leupeptin effectively terminated this tension drop, demonstrating that the tryptic treatment was specific for the digestion of titin/connectin. The steady level of resting tension, which was measured after halting the protease reaction at 2 min of tryptic treatment of myofibrils, showed a SL dependency (Fig. 6C). Moreover, Aw showed an overall increase after 2 min of tryptic treatment (Fig. 6D). Although the absolute value of Aw increased over a range of SL from 2.2 µm to 3.0 µm, a trend of Aw decreasing with the SL was maintained (Fig. 6D).

### Effects of tryptic treatment on SPOC

**Figure 7:**
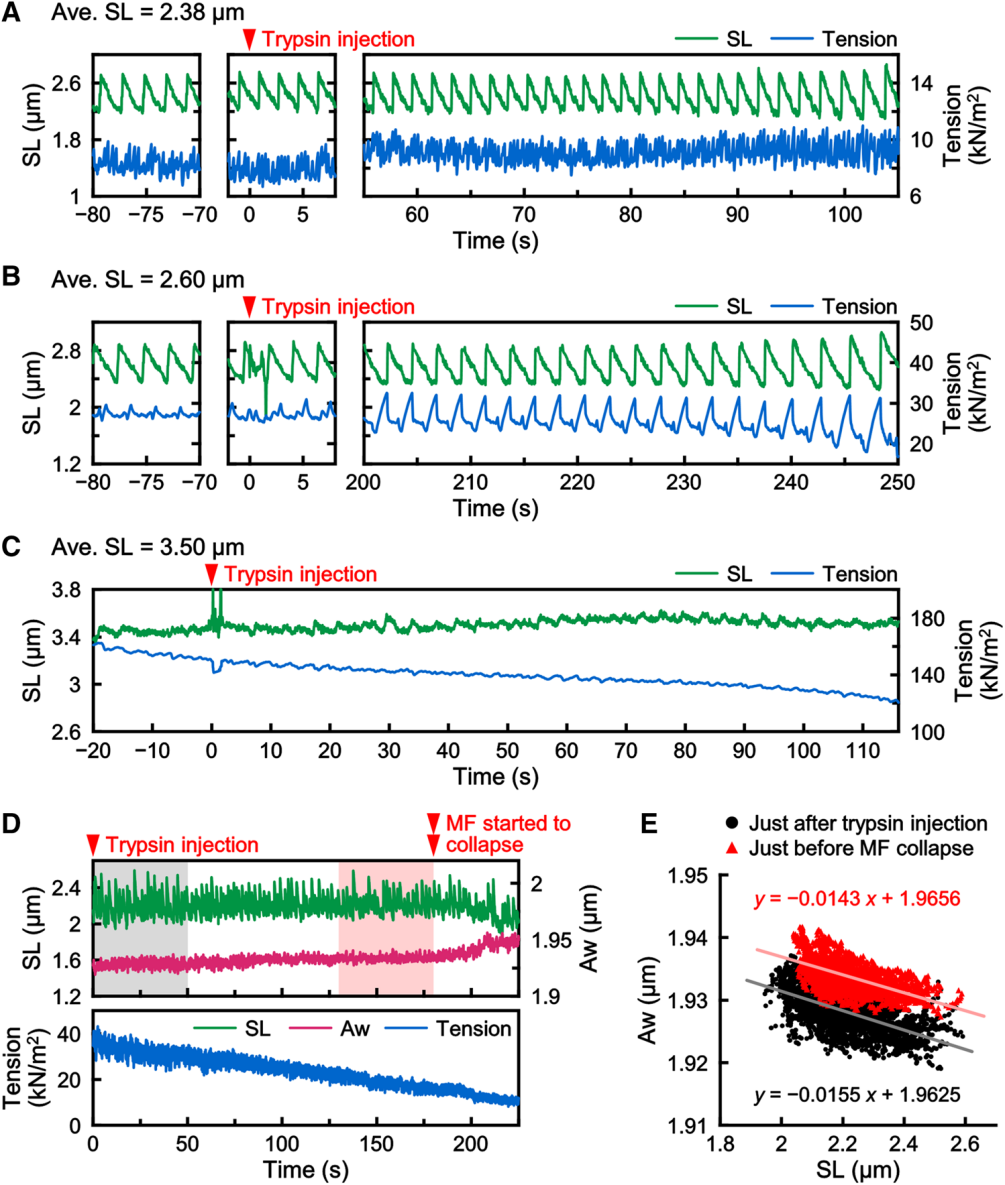
Effects of tryptic treatment on the oscillations of SL, tension and Aw. The ends of myofibrils were held with glass microneedles to examine the effects of trypsin on SPOC. (**A–C**) Time courses of the changes in SL (green) and tension (blue) at average SLs 2.38 µm (**A**), 2.60 µm (**B**) and 3.50 µm (**C**). Trypsin (0.10 µg/ml) was introduced at 0 s as shown by a red arrowhead. Three panels in (**A**,**B**) are SPOC patterns observed before the tryptic treatment (left), at the timing of trypsin injection (center; *t* = 0 s), and over the period until myofibrils were degraded [right; *t* = 105 s for (**A**) and *t* = 250 s for (**B**)]. In the measurement at average SL 3.50 µm (**C**), the myofibril was finally cut near the glass needle and detached from the needle. (**D**) Time courses of the changes in SL (green), Aw (pink) and tension (blue). Trypsin (0.10 µg/ml) was introduced at 0 s (a red arrowhead). The myofibril began to collapse at 180 s (a red double arrowhead). (**E**) Correlation between the changes in the SL and the Aw within the sawtooth wave form in SPOC, of which data were taken from (**D**). The black plots were taken immediately after trypsin injection [the gray region in (**D**)], and the red plots were taken just before the myofibril collapsed [the pink region in (**D**)]. The equations and solid lines (light colors) were obtained by linear regression analysis.

We finally examined the effect of trypsin on SPOC (Fig. 7). We found that the tryptic treatment did not significantly affect the period and amplitude of SL oscillation over several min (Fig. 7, red arrowheads), especially, when the average SL was relatively short (Fig. 7A for ~ 2.4 µm). As the contribution of titin/connectin is expected to be small at this SL, we also examined the consequence at intermediate SL (Fig. 7B for ~ 2.6 µm). We found that the tension gradually decreased after the addition of trypsin, which was most likely due to a partial cleavage of titin/connectin; however, the period and amplitude of the SL and tension oscillations did not change significantly. At an extremely long SL (Fig. 7C for ~ 3.5 µm), the SL oscillation almost disappeared even before the tryptic treatment, probably because the resting tension generated by the extension of titin/connectin may have overcome the active contractile force generated by cross-bridges^8^. Importantly, the Aw oscillation was maintained in the presence of trypsin as long as the sarcomere oscillation was observed (Fig. 7D), although the baseline of the Aw oscillation gradually increased from ~ 80 s after the treatment, and then markedly increased from ~ 180 s just before the myofibril structure became disorganized. The correlation between the changes in SL and Aw showed that Aw became wider upon tryptic treatment, but the slopes of the SL versus Aw correlation were almost equal (Fig. 7E). It is to be noted that SPOC occurs even at the short SL range where titin/connectin has only a minor contribution to the generated tension. Together, these suggest that (1) the titin/connectin is not required for the occurrence of SPOC, and (2) the absolute value of the myofilament lattice spacing is not essential but its negative correlation with SL is important.

## Discussion

Our high-resolution microscopy assay has quantitatively revealed the dynamic changes in the three-dimensional structure of oscillating sarcomeres in SPOC and their sensitivity to physical (i.e., dextran) and biochemical (i.e., trypsin) perturbations. In particular, our data, showing the anti-phase oscillation between the Aw and SL (Fig. 2), are consistent with the prediction drawn from our theoretical model^17–19^ and provide rigorous experimental evidence that the change in the filament lattice spacing is involved in SPOC. It has been widely recognized that the volume of relaxed intact muscle fibers is maintained nearly constant (for a review, see Ref.^58^), by which the cross-sectional area, and thus the lattice spacing of sarcomeres, decreases with increasing SL. We have demonstrated that this volumetric effect can also be observed in sarcomeres of skinned myofibrils, although a constant volume is not strictly maintained (Fig. 6D; cf. Ref.^21^). It has been recognized that titin/connectin contributes to the SL-dependent change in the filament lattice spacing^38–41^, but our analysis revealed that the anti-phase oscillation occurs even at a relatively short SL at which the contribution of titin/connectin (i.e., resting tension) is considerably small (Fig. 6C). Furthermore, the anti-phase oscillation did not cease even when the predominant fraction of titin/connectin was enzymatically digested (Fig. 7E). Taken together, we predict that the Aw change that occurs with SL change is a property that is intrinsic to the lattice structure of sarcomeres with no major involvement of titin/connectin. Possible reasons for the Aw change associated with sarcomere shortening include electrostatic forces that act between myofilaments^10,38,58^, viscoelasticity derived from cross-bridges (for the elastic constant, see Ref.^59^), and the mechanical properties of the Z-disks and the M-bands (e.g., Refs.^57,60^).

The amplitude of Aw oscillation (e.g., 4–6%; Fig. 3), which we experimentally determined using tracer microbeads attached to the sides of single sarcomeres, suggests how the filament lattice spacing controls the probability of cross-bridge formation. For individual sarcomeres to maintain stable SPOC oscillation, the contractile force exerted at the longest SL must be larger than the force generated at the shortest SL. This requires that the probability of cross-bridge formation increases with increasing SL, as the overlap between the thick and thin filaments, which defines the number of available myosin heads that can interact with the thin filaments, decreases with increasing SL. In a typical SPOC condition, the extent of filament overlap can change by 1.4- to 1.7-fold associated with the change in SL (Figs. 3A top, 7A). Here, the ~ 5% of the Aw oscillation amplitude that we experimentally determined corresponds to 1.2 nm change in the distance between the thick and thin filaments (= 23 nm × 0.05, where the distance between the two adjacent thick filaments is assumed to be 40 nm). Because the distance between the surface of the two myofilaments is estimated to be 12 nm (= 23 − 3.5 − 7.5 nm, where 3.5 nm and 7.5 nm are the radii of the thin and the thick filament cores, respectively), the 1.2 nm change corresponds to 10% change in the surface distance. In our theoretical model^17^, the 10% decrease in the surface distance can promote the cross-bridge formation to the extent that is sufficient to compensate for the reduction in the number of available myosin heads within the filament overlap that linearly decreases with SL, providing a quantitative support for that the Aw change is essential for SPOC.

Skinned muscle fibers and myofibrils have a filament lattice spacing that is wider than intact fibers because of the absence of membrane systems. Our dextran experiments, osmotically compressing the filament lattices of skinned muscle myofibrils, showed that the sarcomeres can maintain their oscillatory properties with a narrower lattice spacing, suggesting that SPOC can occur in a physiologically relevant (non-swollen) condition (Fig. 4).

Another important characteristic of SPOC is the propagation of the oscillatory phase between adjacent sarcomeres (i.e., the travelling wave). We previously showed that adjacent sarcomeres in a myofibril can coordinate their force-generating state via a structural change in the Z-disk^2,54^. Our model^18,19^ supports this result, recapitulating the propagation of the oscillatory wave associated with the structural change of the Z-disk. The change in the Z-disk structure might occur due to a change in the filament lattice spacing, i.e., the lateral expansion and compression of the filament lattices. However, we should also consider the contribution of the torque component of cross-bridges. For example, an in vitro motility assay demonstrated that actin filaments gliding over a myosin-coated coverslip surface can rotate around their long axis^61,62^ and form a superhelix^63^. Also, the actin filament can undergo a rolling sliding motion in sarcomeres^64,65^. The torque may cause the generation of torsion in the myofilaments as sarcomeres contract. This torsional strain may induce a structural change in the Z-disk, and the release of the strain associated with detachment of cross-bridges in a sarcomere may propagate toward adjacent sarcomeres, and spread across the myofibril^66,67^. In the future, such complex filament motion could be implemented in our SPOC model to fully elucidate the molecular-level dynamics of oscillating sarcomeres.

Our data also suggest that titin/connectin is dispensable for SPOC, but it might have a minor role in modulating the sarcomere’s oscillatory properties (Fig. 7). This is because the period of SL oscillation, as well as it’s amplitude and waveform, were all maintained nearly indistinguishable from before tryptic treatment (Fig. 7A–C). The effect of tryptic digestion to SPOC appeared only when the myofibril structure started to disorganize (Fig. 7D). Interestingly, we observed that the absolute value of Aw gradually increased at the onset of trypsin treatment (Fig. 7E). On the other hand, the anti-phase oscillation between Aw and SL was maintained just before the myofibril started to disorganize. We predict that the primary role of the titin/connectin is to determine the absolute position of the myofilament lattice spacing, as previously proposed by Horowits and Podolsky^68^, while leaving the dependence of the lattice spacing on SL.

**Figure 8:**
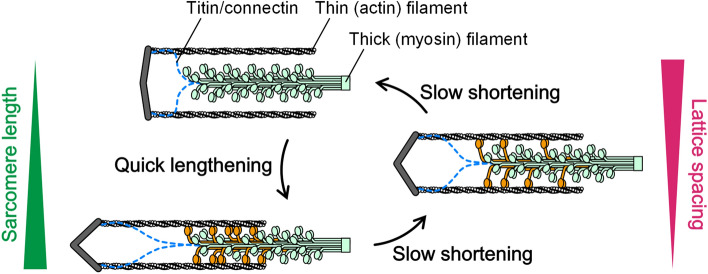
Schematics showing the changes in the sarcomere structure during SPOC. For simplicity, the unit lattice structures of a sarcomere are depicted with one thick filament (green) and two thin filaments (gray). As the sarcomere shortens, the number of available myosin heads increases with the filament overlap (from bottom left to middle right panel). However, the interaction between the thick and thin filaments becomes weaker as the filament lattice expands and the cross-bridges formed between the two filaments tend to be detached by a lateral expansive force (middle right panel). Once the majority of cross-bridges is disengaged from the thin filaments (top left panel), the sarcomere will be unable to support an applied load and quickly lengthens. This leads to the filament lattice becoming narrower, promoting the formation of cross-bridges (bottom left panel). Orange-colored myosin heads indicate cross-bridges formed. Dotted lines represent titin/connectin, whose contribution to SPOC and Aw change appeared not to be essential but had a minor role in modulating the positions of the filament lattice.

Based on our results, we propose how the steady sarcomere oscillation is generated and maintained in SPOC (Fig. 8). The oscillation occurs as a result of the contractile force exerted by myosin motors pulling actin filaments, whose activity changes with the lattice spacing of the sarcomere. Myosin motors form cross-bridges on actin filaments and slide past each other by harnessing the energy of ATP, leading to the shortening of the sarcomere (lower left to middle right schematics, Fig. 8). This results in the filament lattice spacing to become widened, spatially limiting the interaction of motors with the filaments and thus suppressing the probability of cross-bridge formation (middle right schematic, Fig. 8). The reduced number of cross-bridges renders the sarcomere to be mechanically unstable and the two filaments to be disengaged upon thermal noise or pulling force from other sarcomeres (upper left schematic, Fig. 8). The dissociated cross-bridges can then rebind to the actin filament as the sarcomere lengthens and the lattice spacing narrows (lower left schematic, Fig. 8).

Although our model can recapitulate the essential SPOC characteristics with experimental evidence, several questions remain. Firstly, the function of the M-band, which is located at the center of the bipolar thick filament in each sarcomere, should be determined. Increasing evidence suggests that a structural change in the Z-disk mediates the propagation of the SPOC wave between adjacent sarcomeres^2,54^, but the mechanism by which the wave propagates across adjacent half-sarcomeres in each sarcomere is not known. Secondly, it is interesting to consider whether the Super-Relaxed State (SRX)^69^, which has been attracting attention in recent years as a new relaxed state of myosin heads, is involved in the SPOC mechanism. As the timescale of SRX is on the order of several minutes to hours, we predict that the involvement in SPOC is not for each shortening-lengthening oscillation of sarcomeres (period: seconds) but rather for a long-term suppression of the developed force, if any. Finally, the myosin binding protein C (MyBP-C), which is located around the center of sarcomeres and regulates the interaction between the thick and thin filaments, may play a role in SPOC. Importantly, Napierski et al*.* recently reported that the phosphorylation of MyBP-C is important for the occurrence of SPOC (cf. Ref.^70^). To fully understand the muscle mechanics in SPOC it will be necessary to determine whether the phosphorylation merely suppresses the overall level of force generation, or it locally regulates the activity of individual myosin heads along the thick filament.

In summary, our data suggest that the nanoscopic change in the three-dimensional lattice structure of sarcomeres is central to the mechanism of steady SPOC oscillation. The striated muscle is a system in which motors use chemical energy to generate active mechanical force. Our study demonstrates that motors also change the size of the nanoscopic reaction space in which they operate, and feed the altered structural information back to its own activity in an autocatalytic manner, thereby self-controlling the oscillation. The muscle sarcomeres are liquid-crystalline-like structures (cf. Ref.^10^). The precise description of sarcomere behaviors, as well as the knowledge of active and soft matter physics, will lead to a comprehensive understanding of this essential biological machinery.

## Supplementary information

Supplementary Information 1

Supplementary Figure 2

Supplementary Video 1

## Data Availability

The datasets generated for this study are available on request to the corresponding authors.
