# Supplementary material for: Nanoscopic changes in the lattice structure of striated muscle sarcomeres involved in the mechanism of spontaneous oscillatory contraction (SPOC)
Source: Sci Rep. 2020 Oct 2;10:16372. doi: 10.1038/s41598-020-73247-1 (PMC7532212; doi:10.1038/s41598-020-73247-1)
Supplement: Supplementary file 1 — Supplementary Information 1 [file 41598_2020_73247_MOESM1_ESM.pdf]

Supplementary information

Nanoscopic Changes in the Lattice Structure of Striated Muscle Sarcomeres Involved in the Mechanism of Spontaneous Oscillatory Contraction (SPOC)

Fumiaki Kono[1,2,†], Seitaro Kawai[1,†], Yuta Shimamoto[3,*] & Shin'ichi Ishiwata[1,*]

[1] Department of Physics, Faculty of Science and Engineering, Waseda University, 3-4-1 Okubo, Shinjuku-ku, Tokyo 169-8555, Japan

[2] Institute for Quantum Life Science, National Institutes for Quantum and Radiological Science and Technology, 2-4 Shirakata, Tokai-mura, Naka-gun, Ibaraki 319-1106, Japan

[3] Laboratory of Physics and Cell Biology, Department of Chromosome Science, National Institute of Genetics, 1111 Yata, Mishima, Shizuoka 411-8540, Japan

* Correspondence: ishiwata@waseda.jp (S.I.), yuta.shimamoto@nig.ac.jp (Y.S.)

† Equal contributions

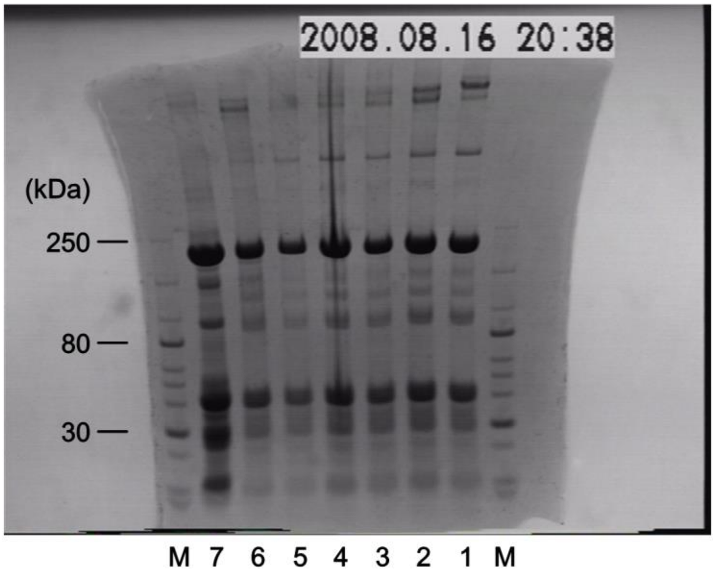

Figure S1. SDS-PAGE gel image of trypsinized muscle fibers.

M: Molecular weight marker, lane 1: untreated, lane 2: treated for 2 min, lane 3: treated for 4 min, lane 4: treated for 8 min, lane 5: same as lane 4 but half the amount was loaded, lane 6: treated for 10 min, lane 7: treated for over 10 min. Lanes 1–4 are displayed in Fig. 6A.

Movie S1

Phase-contrast images showing ADP-SPOC of skeletal myofibril on which both lateral sides of A-band in a sarcomere a pair of plastic micro-beads were attached with the use of optical tweezers. The movie was taken at a video rate (30 fps) and the analysis results of the oscillation waveforms of SL and Aw are shown in Fig. 2A. Scale bar, 2 μm.
