# Supplementary figures and images for: Nanoscopic changes in the lattice structure of striated muscle sarcomeres involved in the mechanism of spontaneous oscillatory contraction (SPOC)
Source: Sci Rep. 2020 Oct 2;10:16372. doi: 10.1038/s41598-020-73247-1 (PMC7532212; doi:10.1038/s41598-020-73247-1)

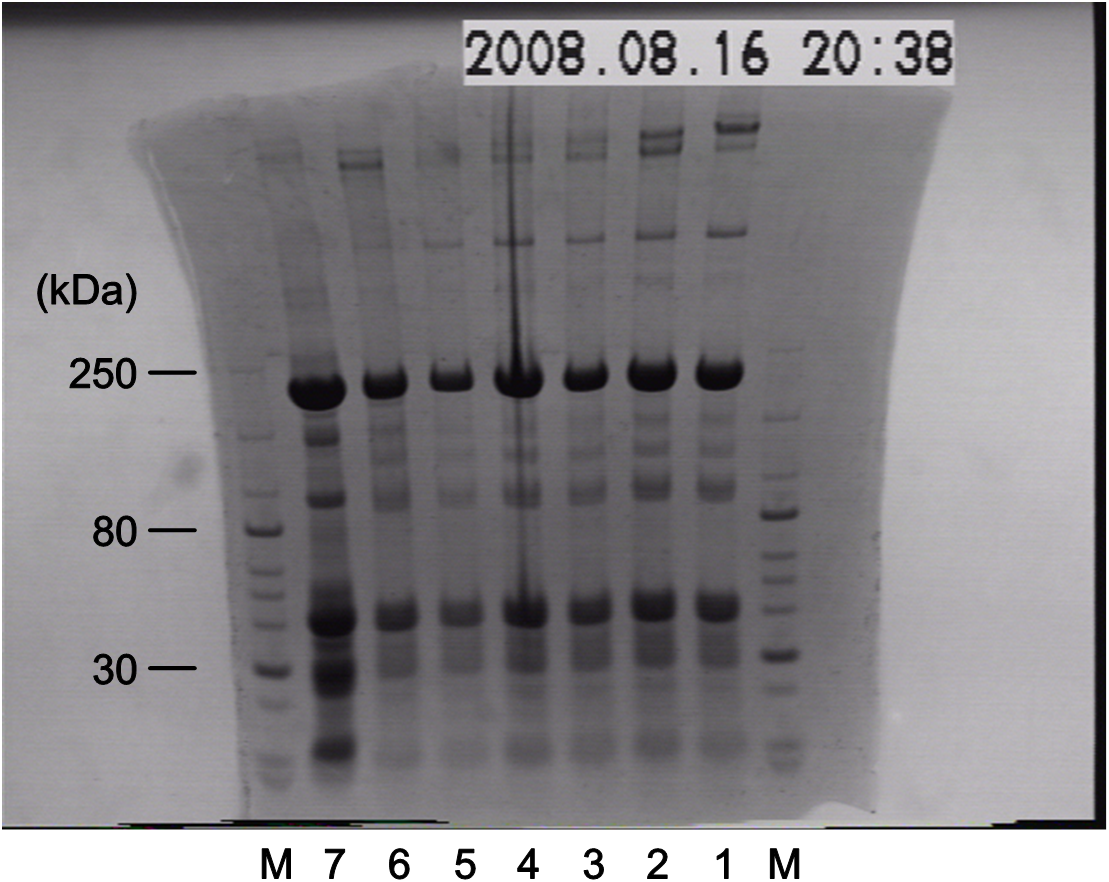

Supplement: Supplementary file 2 — Supplementary Figure 2 [file 41598_2020_73247_MOESM2_ESM.tif]
